# COVID-19, domestic violence and abuse, and urgent dental and oral and maxillofacial surgery care

**DOI:** 10.1038/s41415-020-1709-1

**Published:** 2020-06-26

**Authors:** Paul Coulthard, Iain Hutchison, Joseph A. Bell, Imogen D. Coulthard, Helena Kennedy

**Affiliations:** 1 Dean & Director, Professor of Oral & Maxillofacial Surgery, Institute of Dentistry, Consultant in Oral Surgery, Bart’s Health NHS Trust, Barts and The London School of Medicine and Dentistry, Queen Mary University of London, Turner Street, London, E1 2AD, UK; 2 Professor of Oral & Maxillofacial Surgery, Queen Mary University of London, Director, The National Facial Oral and Oculoplastic Research Centre (NFORC), Chief Executive Saving Faces – The Facial Surgery Research Foundation, UK; 3 Dental Core Trainee, Oral and Maxillofacial Surgery, Bradford Teaching Hospital NHS Foundation Trust, UK; 4 RISE Charity DVA Ambassador Brighton, Biomedical Science Student, University of Brighton, UK; 5 Doughty Street Chambers, Director of the International Bar Association’s Institute of Human Rights, UK

## Abstract

Household isolation measures to reduce coronavirus transmission during the COVID-19 pandemic have resulted in increased risk of domestic violence and abuse (DVA). DVA physical injury most frequently involves the face. Dentists, dental care professionals, oral surgeons and oral and maxillofacial surgeons all have a critical part to play in identifying patients experiencing DVA, who present with dental and facial injury, and in making referrals to specialist agencies. This paper describes how to ask questions about DVA sensitively and how to make an appropriate referral. Early intervention and referral to a DVA advocate can prevent an abusive situation becoming worse with more intense violence. It can save lives.

## Key points

- Description of the role of the dental team in domestic violence and abuse (DVA).
- Advice on identification of DVA in trauma history.
- Guidance on referral of patients disclosing DVA.

## Introduction

Measures have been introduced in many countries during the COVID-19 pandemic to suppress the transmission of coronavirus in the community by reducing contact between individuals and imposing household isolation. These measures drastically affect most people's day-to-day life, but for those experiencing domestic violence and abuse (DVA), there are increased risks. This has been acknowledged by the British government, and others.^1^ The British government have made clear that the household isolation instruction does not apply to those who need to leave their home to escape domestic abuse. The Home Secretary committed additional funding to boost charities' online services and helplines. Domestic abuse is unacceptable in any situation, no matter what stresses people are under, including restricted movement, job loss and financial pressure. DVA is a violation of human rights with short- and long-term damage to physical and mental health. Children are particularly at risk in the 'pressure cooker' of family life in isolation without the usual external oversight of teachers, general medical practitioners and others.^2^ A reported increase of 22% in supermarket sales of alcohol in the UK during March 2020 also highlights the risk.^3^ There has been international recognition of the unintended negative consequences of the COVID-19 global pandemic management measures, including a spike in DVA along with psychological health risks, loneliness, school closure, economic vulnerability and job losses.^4^^,^^5^^,^^6^^,^^7^^,^^8^

DVA is described as a pattern of behaviour involving violence or other abuse by one person against another in a marriage, cohabitation or between family members.^9^ An estimated 2.0 million UK adults aged 16 to 59 years experienced domestic abuse in the year ending March 2018, equating to a prevalence rate of approximately 6 in 100 adults. Women were around twice as likely to experience domestic abuse as men (7.9% compared to 4.2%).^10^ DVA is more than physical violence; it can also include, but is not limited to, coercive control and 'gaslighting', economic abuse, online abuse, verbal abuse, emotional abuse and sexual abuse. Physical violence is generally more severe against women than against men.

When DVA involves physical assault, the face is a very common target, with studies suggesting that between 65% and 95% of assaults involve trauma to the face.^11^^,^^12^ Consequently, the dentist, dental care professional, oral surgeon and oral and maxillofacial surgeon all have a significant part to play in identifying DVA.^13^^,^^14^

Many countries were unable to contain the spread of the coronavirus and adopted various strategies to reduce the contagion, in order to prevent their health services being overwhelmed. Most countries have reduced routine dental care to avoid virus transmission and only carry out urgent dental procedures.^15^^,^^16^ In the UK, access to urgent care is via telephone or video triage to avoid unnecessary contact. It is important that this consultation, along with any subsequent patient attendance for urgent dental care, is used as an opportunity for identification of DVA in patients reporting dental or facial injury. Frontline oral and maxillofacial surgeons continue to see patients who attend accident and emergency (A&E) departments presenting with dental and more severe facial injury, and they too are therefore key to identifying DVA. Dental professionals are well placed to observe and identify injuries to the head, eyes, ears, neck, face, mouth and teeth. Bruising, burns and bite marks are types of injury that should suggest concern.^17^

Some dentists and healthcare professionals may not consider that identification of DVA is their responsibility because they assume that social services or the police will be doing something.^18^ However, we know that other services may not have been approached and, even if they have, it is better for someone to be asked on numerous occasions than not asked at all.^19^ Social and youth workers and safeguarding teams have been depleted as staff have needed to self-isolate. The 'stay at home' message has also been accompanied by a fear of contracting COVID-19 on attending healthcare providers, such that the UK has seen a reduction in patient attendance at general medical practices and a 25% reduction in attendance at A&E departments.^20^ This highlights the importance of maximising the opportunity to identify DVA afforded to the dental team when patients do present.

Dentists and dental care professionals may be anxious about asking for the cause of a dental or facial injury; they are often not confident in asking what they may feel are invasive questions about DVA, nor do they know what to do should their patient disclose.^18^ Undergraduate education in the UK may not have prepared them for such a sensitive inquiry and postgraduate training is not widely available.^21^ It has also been reported that oral and maxillofacial surgery (OMFS) frontline staff may lack the confidence for such a personal exchange. Both groups therefore need training to develop knowledge and build confidence.^12^^,^^22^ Brief intervention training can increase knowledge and change attitudes.^23^ Challenging stigma, myths and stereotypes can be an important part of training. Confidence to ask DVA questions appropriately can be more effectively acquired through interactive learning and practice than from reading this paper. This article will, however, provide some educational support to those yet to access training by providing suggested questions that could be incorporated into the trauma history and providing information on making a referral to an appropriate DVA agency.

## Dental and OMFS role in DVA

The role of the dental and OMFS team is to 'identify' DVA and 'refer' patients for specialist help.^24^ It is not their role to provide advice and this could be dangerous. Well-intentioned but ill-informed advice, such as to leave the abusive relationship, can be positively dangerous. Women who leave their partners can face an increased risk of assault.^25^ Dental and OMFS teams should facilitate contact with appropriate local services. Some may think that this role is more appropriate for the general medical practitioner rather than themselves, not recognising the importance of their own role and the opportunities that there are.

## Telephone/video triage during the COVID-19 pandemic

Any UK patient requesting urgent care is telephone/video triaged by a dentist to assess their clinical urgency, establish their COVID-19 risk, offer any interim self-care advice or arrange a face-to-face assessment as appropriate. The first and foremost concern is for the safety of the patient when asking about the aetiology of trauma. If the patient is with the perpetrator, then disclosure will not be possible and would place the woman or man in greater danger. When enquiring about aetiology, ask the patient to respond with 'yes' or 'no' to whether it is safe to talk. If the answer is 'no', then ask whether she or he is feeling safe or in danger. If the patient is in immediate danger, offer to call the emergency services using 999 in the UK. The patient can also phone the emergency number 999 and then press 55 if from a mobile, which will transfer the call to the relevant police force, who will assist without the patient having to speak. Pressing 55 lets the operator know that the call is genuine. A landline 999 call provides location information to the call handler. If the aetiology of the trauma is disclosed as DVA, then ask if children are present as an immediate safeguarding referral should be made, even if the emergency services are not required as there is no immediate danger.

The written record of the telephone consultation should include a note of any disclosure or any suspicion of DVA. The British government includes those registered with the General Dental Council among 'appropriate health professionals' for the provision of evidence to a court.^26^ Clinical photographs of the injury will be useful.

Evidence can also be important in helping an abused woman to obtain protection through an injunction or court order, or in opposing an immigration or deportation case. It can be used by the family courts to assess possible risks in granting access to children to a violent parent.

## The urgent dental care environment

It is useful to take advantage of working in the dental or A&E environment, which is likely to be considered less stigmatising than some other statutory services, to create an opportunity for the patient to reveal domestic violence and ask for help.

As for a telephone consultation, there needs to be consideration that the presence of a partner or a relative may constrain discussion of DVA. Asking the woman/man about domestic violence could place the woman or man in greater danger if the perpetrator is present. If the patient does not speak good English, her/his abuser may try to play the role of interpreter to inhibit any disclosure. However, the dental environment offers a unique and advantageous position to undertake inquiry about DVA, as patients are often unaccompanied during examination and treatment sessions as part of routine practice. It is important that all the dental team are aware of the need for a safe environment to permit DVA disclosure, as should all A&E staff. It may be possible to divert the partner to the reception for the completion of documentation without arousing suspicion.

## Asking about domestic violence

The dental and OMFS team should be confident and supportive in their approach, expressing concern as necessary without accusation or being patronising. A non-judgemental tone of voice and body language are essential to permit disclosure. Vague enquiries are not helpful. The appropriate time to ask is when enquiring about the cause of the injury - how, when and where it occurred. Direct questions should be asked as usual practice when taking a history. Sometimes, however, it might be helpful to explain why a question is being asked by explaining the following: 'I am sorry if someone has already asked you about this, and I don't wish to cause you any offence, but we know that throughout the country one in four women/one in six men experiences violence at some time during their life and so we are asking all women/men about this issue'.^27^

Unfortunately, the victims, when confronted with a clinician mentioning the possibility of domestic violence, often vehemently deny the possibility because of anxiety about further violence or the state taking their children away for safety reasons. In this situation, the clinician may be at a loss to know how to report their suspicions.

DVA occurs in all socioeconomic groups, all ages and all races; therefore, all patients should be given the opportunity to disclose. If the description of the dental or facial injury does not appear to be consistent with the stated aetiology, then this should be explained to the patient. If the patient has multiple injuries or appears frightened or excessively anxious, then this may be suggestive of DVA.

Questions such as: 'Is everything alright at home?' or 'Are you ever afraid at home?' may be useful to assess risk before asking the direct question: 'Do you feel safe or in danger?' In the urgent care or A&E department setting, the patient can be told that they are in a safe space. A single question may not provide sufficient time for a patient to consider disclosure and a further different question or two may permit this. Further questions are suggested in Box 1.

Confidentiality is essential as patient safety may be dependent on this being maintained. However, it is important to understand and be honest about the limits to confidentiality. If there is a disclosure of DVA and the presence of children under 18 years of age in the household is admitted, then protection of the children takes precedence over patient confidentiality. Similarly, if the victim in question is pregnant or there are vulnerable adults living in the household, a disclosure of DVA should trigger a safeguarding referral whether the victim consents or not.

Box 1 DVA disclosure example questions (please use after reading the text of this paper) The following questions are intended as prompts; it will not be necessary or appropriate to ask all of these:

- Could you tell me how you got your injury?
- Was your injury caused by someone you know?
- Your partner seems very concerned and anxious about you. Sometimes people react like that when they feel guilty; were they responsible for your injuries?
- Do you ever feel frightened of your partner or other people close to you?
- I am sorry if someone has already asked you about this, and I don't wish to cause any offence, but we know that throughout the country one in four women/one in six men experiences violence at home at some time during their life. I noticed that you have a number of... (bruises/cuts, as appropriate)
- Have you ever been slapped, kicked or punched by your partner?
- Does your partner often lose their temper with you? If he/she does, what happens?
- Has your partner ever:
  - Destroyed or broken things you care about?
  - Threatened or hurt your children?
  - Forced sex on you, or made you have sex in a way you did not want?
  - Withheld sex or rejected you in a punishing way?
- Does your partner get jealous of you seeing friends, talking to other people or going out? If so, what happens?
- Does your partner use drugs or alcohol excessively? If so, how does he/she behave at this time?

## Referral

The purpose of identification of domestic violence is referral to the appropriate agency and so this pathway must be considered before making the inquiry. There are many national and local agencies whose purpose is to provide professional risk assessment, advice and support. Table 1 shows the details of some national agencies. Many are charities, some supported by government; they work with the police and social services or provide online support, helplines and refuges. It is not the role of the dentist or oral and maxillofacial surgeon to offer advice on how a patient should manage DVA, but it is their role to make a referral to an advocate. Referral may be made by telephoning an agency and arranging an appointment or by forwarding a written referral online. Alternatively, it may be by providing the patient with a contact phone number so that the patient may arrange directly at a safe time. This number may be provided on a discrete small card that can be hidden on the person; for example, in a shoe. These are made available by most agencies on request and in a range of languages as appropriate. Most of these telephone numbers do not show up on telephone invoices, so cannot alert a watchful abuser to a victim's attempts to seek help. Those working in hospital OMFS are likely to have a protocol with a pathway for referral to an on-call safeguarding lead and requirement to discuss with a senior member of staff within the team or A&E team.

**Table 1:** Some UK national referral agencies

Agency	Description	Contact
UK Government	Support for victims of domestic abuse during COVID-19	Website: https://www.gov.uk/government/publications/coronavirus-covid-19-and-domestic-abuse/coronavirus-covid-19-support-for-victims-of-domestic-abuse
National Domestic Abuse Helpline (managed by Refuge)	Supporting women	Website: https://www.nationaldahelpline.org.uk Phone number: 0808 2000 247
Men's Advice Line	Supporting men	Website: https://mensadviceline.org.uk Phone number: 0808 801 0327
Galop	Supporting members of the LGBT+ community	Website: https://www.galop.org.uk/galop-to-run-national-lgbt-domestic-violence-helpline/ Phone number: 0800 999 5428
Karma Nirvana	Supporting victims of 'honour'-based abuse	Email: support@karmanirvana.org.uk Phone number: 0800 5999 247

It can be useful for DVA posters to be displayed in surgery waiting rooms or toilets with contact telephone numbers for urgent care, or they can be put up in A&E departments. These can provide a signal to encourage patients to disclose DVA to a health professional.

## Summary

If the dental and OMFS team only focus on treating injuries, without asking about their cause, then they will be doing little to help the patient who is experiencing DVA. The patient should be asked about the cause of their injury and appropriate further questions as a follow-up. This offers the opportunity for disclosure of the DVA if it was not mentioned initially. Patients should be supported in any disclosure that they make and there should then be an assessment of their risk. A referral should take place to a local or national DVA service that can offer professional risk assessment and support. It is crucial that the dental and OMFS team engage. Early intervention and referral to a DVA advocate can prevent an abusive situation becoming worse with more intense violence. It can save lives.
